# Validation of prognostic scoring and assessment of clinical benefit for patients with bone sarcomas enrolled in phase I clinical trials

**DOI:** 10.18632/oncotarget.10910

**Published:** 2016-07-28

**Authors:** J. Andrew Livingston, Kenneth R. Hess, Aung Naing, David S. Hong, Shreyaskumar Patel, Robert S. Benjamin, Joseph A. Ludwig, Anthony Conley, Cynthia E. Herzog, Pete Anderson, Funda Meric-Bernstam, Razelle Kurzrock, Vivek Subbiah

**Affiliations:** ^1^ Division of Cancer Medicine, The University of Texas MD Anderson Cancer Center, Houston, Texas, USA; ^2^ Department of Biostatistics, The University of Texas MD Anderson Cancer Center, Houston, Texas, USA; ^3^ Department of Investigational Cancer Therapeutics (Phase I Clinical Trials Program), The University of Texas MD Anderson Cancer Center, Houston, Texas, USA; ^4^ Department of Sarcoma Medical Oncology, The University of Texas MD Anderson Cancer Center, Houston, Texas, USA; ^5^ Department of Pediatrics, The University of Texas MD Anderson Cancer Center, Houston, Texas, USA; ^6^ Pediatric Hematology Oncology, The Cleveland Clinic, Cleveland, Ohio, USA; ^7^ Experimental Therapeutics Program, University of California – San Diego, San Diego, California, USA

**Keywords:** bone sarcoma, phase 1 trials, prognosis scores, Ewing sarcoma, osteosarcoma

## Abstract

**Background:**

We sought to validate the Royal Marsden Hospital (RMH) and MD Anderson Cancer Center (MDACC) prognostic scoring systems for the selection of bone sarcoma patients for phase I clinical trials and to identify additional risk factors related to survival.

**Patients and Methods:**

We retrospectively reviewed the baseline characteristics and outcomes of 92 bone sarcoma patients who were referred to MDACC's Phase I Clinical Trials Program.

**Results:**

Ninety-two patients with Ewing sarcoma (*N* = 47), osteosarcoma (*N* = 22), chondrosarcoma (*N* = 16), and other tumors (*N* = 7) were evaluated; 78 were enrolled in at least 1 of 43 different phase I trials. The median overall survival (OS) was 8.8 months (95% confidence interval [CI] = 6.8–13.7 months). Independent factors that predicted shorter survival were male sex, >2 metastatic sites, >3 previous therapies, hemoglobin level <10.5 g/dL, platelet count >200 x10^3^/L, creatinine level ≥1.3 mg/dL, and lactate dehydrogenase level >ULN. Patients with good RMH scores (0-1) had longer OS than patients with poor RMH scores (2-3) (HR = 5.8, 95% CI = 2.9–11.0; *P* < 0.0001), as did patients with low MDACC scores (0-1) as compared to patients with higher MDACC scores (2–4) (HR = 3.2, 95% CI = 1.9–5.6; *P* < 0.0001).

**Conclusion:**

The RMH prognostic score can be used to predict the OS of bone cancer patients referred for phase I trials. The MDACC score added no value to the RMH score and therefore does not have a role in assessment of patients with bone tumors. Patients with advanced bone sarcomas should be considered for phase I trials.

## INTRODUCTION

Bone sarcomas are rare tumors, accounting for < 0.2% of all cancers. In the United States in 2014, about 3,000 patients were newly diagnosed with bone sarcomas, and more than 1,400 deaths were attributed to these tumors. [1] Patients with aggressive bone sarcomas, especially patients with advanced, metastatic, or relapsed disease, have an extremely poor prognosis [2, 3]. With the exception of the immunotherapy mifamurtide, which has been approved in the European Union but is not available in the United States [4], no new systemic therapies have been approved for the treatment of the most prevalent bone sarcomas (osteosarcoma, Ewing sarcoma, and chondrosarcoma) in the last 3 decades.

In the United States, cytotoxic chemotherapy remains the standard of care for patients with osteosarcoma or Ewing sarcoma and altering these regimens or increasing their dose intensity has not improved overall survival (OS) for these groups in > 25 years. [5] Similarly, patients with chondrosarcomas, which are resistant to the conventional chemotherapies that are effective against other bone sarcomas, have not seen improvement in their OS in > 30 years. [6] Reasons for the lack of progress in developing effective therapies specifically against bone sarcomas include the rarity of these tumors as well as the complex and heterogeneous tumor biology which make the delivery of targeted therapy even more challenging [7, 8].

Enrolling patients with advanced, relapsed, or refractory bone cancers in phase I trials of targeted therapies may yield some unique insight into previously unexplored therapeutic targets, provide an opportunity to identify signals of early treatment response in these patients, and benefit these patients clinically [9, 10]. However, many clinicians who screen such patients for early-phase clinical trials are often unable to accurately determine the survival profiles of individual patients, quite often an eligibility criterion for the study. Scoring systems that take prognostic factors into account have been established to optimize clinical decision-making regarding patients with advanced cancer. One of these systems, the Royal Marsden Hospital (RMH) scoring system, which is based on 3 survival-associated variables (albumin level, lactate dehydrogenase [LDH] level, and number of metastases), can be used to predict individual patient survival in phase I trials. [11] In a previous study, we validated the RMH scoring system for cancer patients treated at MD Anderson Cancer Center (MDACC). [12] The identification of additional independent prognostic factors for survival in that study led to a modification of the RMH scoring system and the development of the MDACC prognostic scoring system, which considers tumor type and ECOG performance status in addition to albumin level, LDH level, and number of metastases. [13] Both the RMH and MDACC prognostic scoring systems were developed and validated in patients with carcinomas and included few, if any patients with bone sarcomas. Thus, whether these systems can be used to predict outcomes in bone sarcoma patients is unknown. Validating either scoring system for bone sarcoma patients would provide clinicians with a valuable tool for selecting these patients for early-phase clinical trials.

Therefore, the objective of the present study was to validate these predictive models in bone sarcoma patients. We also sought to identify additional risk factors related to OS in this group and determine the clinical benefit these patients derive from participating in phase I trials to better inform physicians who refer bone sarcoma patients for early-phase clinical trial participation.

## MATERIALS AND METHODS

### Patients

We retrieved the electronic records of 92 consecutive patients who were referred to MDACC's Phase I Clinical Trials Program between July 2005 and November 2013 for evaluation for treatment in a phase I clinical trial. We reviewed the patients’ records for the history, laboratory, and clinical findings at the time of presentation to the program, the treatment(s) given, and clinical outcomes. Pathology was reviewed by an MDACC pathologist with expertise in bone sarcomas in all cases. The therapies investigated in the clinical trials varied over time and were dependent on protocol availability at the time of presentation.

We recorded patients’ baseline characteristics at referral to the program, including age, sex, tumor type, Eastern Cooperative Oncology Group (ECOG) performance status, number of prior systemic therapies for metastatic disease, number of sites of metastases, hemoglobin level (g/dL), LDH level (U/L), platelet count (k/uL), and albumin level (g/dL), as well as date of phase I therapy initiation, best response to phase I therapy based on Response Evaluation Criteria in Solid Tumors (RECIST; version 1.1), [14] and date of death or last follow-up. This study was approved by MDACC's Institutional Review Board (IRB). All clinical trials were also reviewed and approved by the institutional IRB. Patients provided their written informed consent to receive treatment on the respective phase I protocols.

### Statistical analysis

Patient characteristics were summarized using medians and ranges for continuous variables and frequencies and percentages for categorical variables.

To test the RMH prognostic scoring system, we classified patients according to 3 variables: LDH level (normal [0] *vs*. > upper limit of normal [ULN; +1]), albumin level (≥3.5 g/dL [0] *vs*. < 3.5 g/dL [+1]), and number of metastatic sites of disease, (≤2 [0] *vs*. > 2 [+1]). The values for each variable were summed to provide a prognostic score, and patients were classified as having a good prognosis (RMH score of 0 or 1) or poor prognosis (RMH score of 2 or 3). To test the MDACC prognostic scoring system, we classified patients according to the 3 RMH variables plus ECOG performance status (0 [0] *vs*. ≥1 [+1]). (Although the MDACC system includes gastrointestinal tumor type, this variable was not included in our analysis, as all patients had bone sarcomas.)

Progression-free survival (PFS) was measured from the time of clinical trial enrollment until imaging demonstrating disease progression by RECIST or last follow-up. OS was measured from the time of presentation to the phase I program until death from any cause or last follow-up. Median OS durations were estimated using the Kaplan-Meier method. [15] Patients were censored at the time of their last follow-up. Univariate Cox proportional hazard analysis was used to compare OS among subgroups of patients.

Univariate and multivariate Cox proportional hazards models were fit to assess associations among patient characteristics and clinical outcomes. [16] The covariates in the univariate analysis included age ( < 40 *vs*. ≥40 years), sex, body surface area ( < 2 *vs*. ≥2 m^2^), tumor type (Ewing sarcoma, osteosarcoma, chondrosarcoma, or other bone sarcoma), ECOG performance status (0, 1, 2, or 3), hemoglobin level ( < 10.5 *vs*. ≥10.5 g/dL), platelet count ( < 200 vs. ≥200 x10^3^/L), albumin level ( < 3.5 *vs*. ≥3.5 g/dL), creatinine level ( < 1.3 *vs*. ≥1.3 mg/dL), number of prior therapies ( < 3 *vs*. ≥3), history of prior radiation (yes *vs*. no), number of metastases (≤2 *vs*. > 2), LDH level (≤618 *vs*. > 618 U/L), RMH score, and MDACC score. These variables were measured at the time of presentation to the phase I program. Variables with *p* > 0.50 were removed from the model due to sample size. Cox proportional hazards analysis was used to validate the RMH and MDACC prognostic scores using our data set. All statistical tests were 2-sided, and *P* values < 0.05 were considered statistically significant. Statistical analyses were conducted with SAS statistical software (version 9.1; SAS Institute Inc., Cary, NC).

## RESULTS

### Patient characteristics

Ninety-two patients (58 men [63%] and 34 women [37%]) with bone sarcomas who were evaluated for participation in phase I clinical trials in MDACC's Phase I Clinical Trials Program were included in this retrospective review. These patients’ baseline characteristics at referral to the program are given in Table 1. The most common tumor type was Ewing sarcoma in 47 patients (51%), followed by osteosarcoma in 22 patients (24%), chondrosarcoma in 16 patients (17%), and other tumors in 7 patients (8%) (chordoma 5, 1 each hemangiopericytoma and malignant chondroid syringoma). The median patient age at referral was 24 years (range, 11-79 years). The median number of prior chemotherapy regimens was 3 (range, 0-11 regimens), and 57 patients (62%) had prior radiation therapy. All patients had either progressed on standard therapy or demonstrated progression in the absence of therapy for tumor types that lack effective standard of care treatments such as chondrosarcoma.

**Table 1:** Baseline characteristics of the 92 bone sarcoma patients in the present study

Characteristic	No. of Patients (%)
Sex	
Male	58 (63)
Female	34 (37)
Age, years	
Median	24
Range	11–79
<18	11 (11)
18–39	53 (58)
40–65	18 (20)
>65	10 (11)
Tumor type	
Ewing sarcoma	47 (51)
Osteosarcoma	22 (24)
Chondrosarcoma	16 (17)
Chordoma	5 (5)
Other	2 (3)
ECOG performance status	
0	25 (27)
1	57 (62)
2	7 (8)
No. of prior chemotherapies	
Median	3
Range	0–11
0–2	47 (51)
≥3	45 (49)
Prior radiation therapy	
Yes	57 (62)
No	35 (38)
Prior immunotherapy, including monoclonal antibodies	
Yes	12 (13)
No	80 (87)
No. of metastases	
≤2	66 (72)
>2	26 (28)
RMH score	
0 or 1	74 (81)
2 or 3	17 (19)
MDACC score	
0 or 1	49 (53)
2–4	40 (45)

RMH: Royal Marsden Hospital prognostic score, MDACC: MD Anderson Cancer Center prognostic score.

### Phase I trials

Of the 92 patients referred for evaluation, 78 participated in at least 1 phase I trial (range, 1-6 trials). Patients participated in 43 different phase I trials, and 10 patients (11%) participated in > 1 trial. Of the 78 patients, 56 (72%) were enrolled in trials that included a tyrosine kinase inhibitor or small-molecule targeted therapy, 33 (42%) were enrolled in trials that included a targeted monoclonal antibody, and 9 (12%) were enrolled in trials that included both a targeted agent and a cytotoxic agent. Most of the trials in which these patients were enrolled included 1-5 bone sarcoma patients total, with the exceptions of trials that included anti-insulin-like growth factor 1 receptor (IGF-1R) antibodies alone (20 patients) or in combination with mammalian target of rapamycin (mTOR) inhibition (11 patients). A representative list of the mechanisms of action of the agents investigated in the clinical trials is shown in Table 2.

**Table 2:** Mechanisms of action of agents in phase I trials enrolling patients in the present study

**Mechanism of action**
Anthracycline + proteasome inhibitor + nucleoside analog
Anti-IL1 monoclonal antibody
Anti-microtubule agent + nucleoside analog + VEGF monoclonal antibody
Aurora kinase inhibitor
Biguanide + mTOR inhibitor
CDK inhibitor
cMet inhibitor
cMET/Alk inhibitor + mulitargeted TKI
Death receptor ligands
EGFR TKI + EGFR monoclonal antibody
HDAC inhibitor + multitargeted TKI
HDAC inhibitor + VEGF inhibitor + mTOR inhibitor
HER2 TKI + biguanide
HIF inhibitor
IgG1/delta3 monoclonal antibody
Immune modulator + mTOR inhibitor
IGF-1R antibody
IGF-1R antibody + mTOR inhibitor
Microtubule stabilizing agent + vitamin K antagonist
mTOR inhibitor
mTOR inhibitor + EGFR monoclonal antibody
mTOR inhibitor + HDAC inhibitor
Multitargeted TKI
PARP inhibitor
PI3K inhibitor
Spliceosome inhibitor
TRAIL receptor 2 antibody
VEGF inhibitor + HER2 TKI
VEGF monoclonal antibody + mTOR inhibitor
VEGF TKI + VEGF monoclonal antibody

VEGF: vascular endothelial growth factor, mTOR: mechanistic target of rapamycin, EGFR: epidermal growth factor receptor, TKI: tyrosine kinase inhibitor, HDAC: histone deacetylase, HIF: hypoxia-inducible factor, IGF-1R: insulin-like growth factor 1 receptor, PARP: poly-ADP ribose polymerase, PI3K: phosphoinositide 3-kinase, TRAIL: TNF-related apoptosis-inducing ligand.

### Clinical outcomes

**Figure 1:**
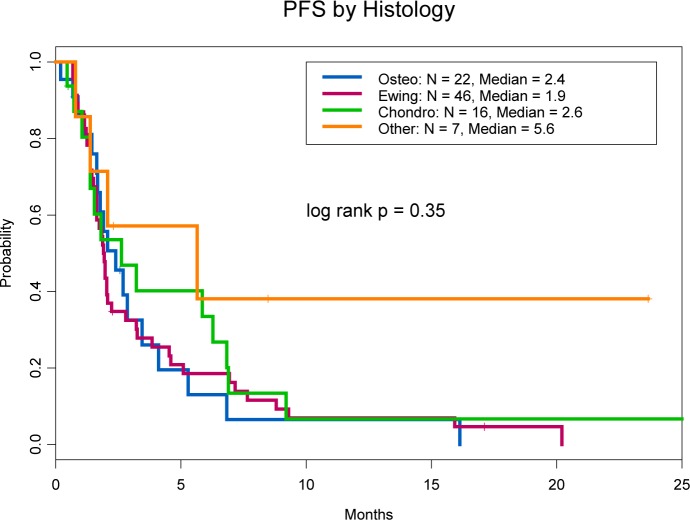
Progression-free survival by tumor type

The median progression-free survival (PFS) for all patients was 2.0 months (95% confidence interval [CI] = 1.8-2.9 months). The 4-month PFS rates were 26% (95% CI = 12-58%) for osteosarcoma patients, 26% (95% CI = 16-42%) for Ewing sarcoma patients, 40% (95% CI = 22-74%) for chondrosarcoma patients, and 57% (95% CI = 30-100%) for patients with other tumor types (Figure 1). The median OS duration of all patients was 8.8 months (95% CI = 6.8-13.7 months); the median OS durations of patients with osteosarcoma, Ewing sarcoma, and chondrosarcoma were 6.1, 8.5, and 13.2 months, respectively.

No patients had a complete response. Five patients (6%) had a partial response, and 24 patients (31%) had stable disease. Disease stabilization > 6 months or partial response was seen in 12 patients (15%). Three Ewing sarcoma patients had responses to anti-IGF-1R therapy with or without mTOR inhibition. Two chondrosarcoma patients had responses to the death receptor ligand therapy Apo2L/TRAIL (recombinant human protein apoptosis ligand 2/TNF-related apoptosis-inducing ligand or dulanermin), with one chondrosarcoma patient remaining on therapy with sustained response for > 5 years. No osteosarcoma patients had a RECIST response. One Ewing sarcoma patient and one chondrosarcoma patient had prolonged disease stabilization for > 20 months and > 13 months, respectively.

### Validation of the RMH and MDACC prognostic scoring systems

Our univariate analysis identified variables associated with shorter OS. Given the modest sample size, variables that were marginally significant in the univariate analysis, including age, ECOG performance status, bilirubin level, and albumin level (*P* > 0.50 for all), were excluded from the multivariate analysis. Variables included in the final model are shown in Table 3. Independent factors that predicted shorter OS in the multivariate Cox model were male sex (hazard ratio [HR] = 2.2, *P* = 0.025), > 2 sites of metastases (HR = 2.6, *P* = 0.0023), > 3 previous therapies (HR = 1.6, *P* = 0.042), hemoglobin level < 10.5 g/dL (HR = 4.4, *P* < 0.0001), platelet count > 200 x10^3^/L (HR = 2.1, *P* = 0.015), and LDH level > ULN (HR = 2.2, *P* = 0.018). Normal renal function (serum creatinine < 1.3 mg/dL) was favorable (HR = 0.4, *P* = 0.024). When included in the model, patients who had prior radiation therapy tended to have longer OS, but this association was not significant (HR = 0.5, *P* = 0.07).

**Figure 2:**
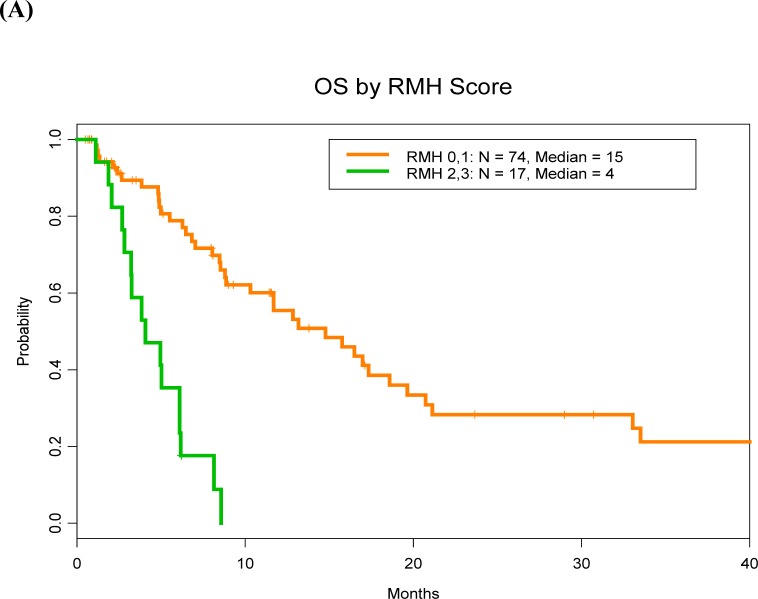

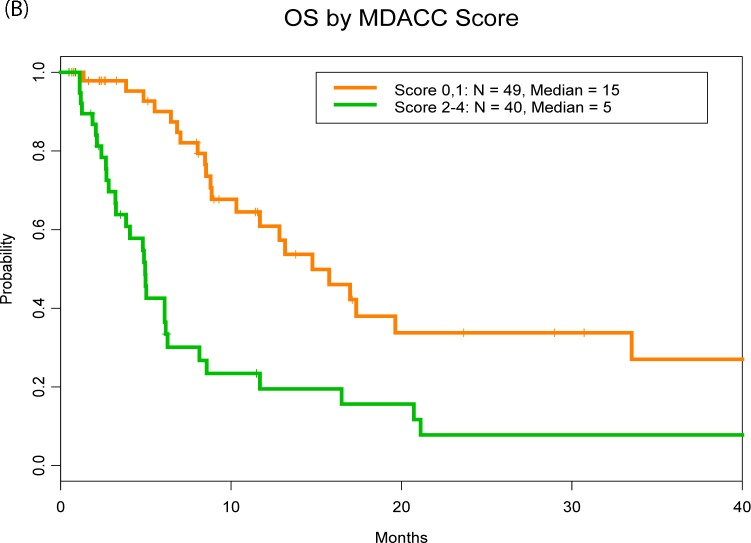
**A.** The median OS duration of bone sarcoma patients who had RMH prognosis scores of 0 or 1 (15 months) was significantly longer than that of patients who had RMH prognosis scores of 2 or 3 (4 months; P < 0.0001). **B.** Similarly, the median OS duration of patients with MDACC prognosis scores of 0 or 1 (15 months) was significantly longer than that of patients who had MDACC prognosis scores of 2-4 (5 months; P < 0.0001)

The median OS duration of bone sarcoma patients who had RMH prognosis scores of 0 or 1 (15 months) was significantly longer than that of patients who had RMH scores of 2 or 3 (4 months; HR = 5.8, 95% CI = 1.9-5.6;*P* < 0.0001) (Figure 2A). Similarly, the median OS duration of patients with MDACC prognosis scores of 0 or 1 (15 months) was significantly longer than that of patients who had MDACC scores of 2-4 (5 months HR = 3.2, 95% CI = 1.9-5.6; *P* < 0.0001) (Figure 2B).

**Table 3:** Multivariate Cox regression model

Cox Model	HR	95% CI	*P*
Ewings *vs*. Osteosarcoma	1.2	0.6, 2.7	0.56
Chondrosarcoma *vs*. Osteosarcoma	0.8	0.3, 2.2	0.7
Other tumor type *vs*. Osteosarcoma	0.1	0.3, 0.6	0.0084
Sex: male *vs*. female	2.2	1.1, 4.4	0.025
Body surface area: ≥2 *vs*. <2	1.7	0.8, 3.4	0.16
Number of metastatic sites: >2 *vs*. ≤2	2.6	1.4, 4.8	0.0023
Number of prior therapies: >3 *vs*. ≤3	1.9	1.0, 3.5	0.042
Hemoglobin: <10.5 *vs*. ≥10.5 g/dL	4.4	2.1, 9.2	<0.0001
Platelets: <200 *vs*.>200 × 10^3^/L	2.1	1.2, 3.9	0.015
Lactate dehyrdogenas (LDH): ≤618 [normal] *vs*. >618 U/L [>ULN]	2.2	1.1, 4.2	0.018
Creatinine: <1.3 *vs*. ≥1.3 mg/dL	0.4	0.1, 0.9	0.024

Note: Age, ECOG PS, Bilirubin, and Albumin were removed from the model due to *p* > 0.50. Adding Prior Radiation to the above model gives HR = 0.5 (0.3, 1.0) with *p* = 0.070.

## DISCUSSION

Our study suggests that the RMH prognostic scoring system can be used help appropriately select bone sarcoma patients for phase I trial participation.

The prognostic variables we identified for bone sarcoma patients are similar to those identified for carcinoma patients. [11] Our analysis revealed that RMH variables, including > 2 metastases and LDH level > ULN, were associated with shorter OS in the Cox model. However, albumin level < 3.5 g/dL was not associated with OS (*P* > 0.50), likely owing to the small proportion of study patients who had low albumin levels (4.4%). In the initial analysis of the RMH scoring system for patients treated in phase I trials at MDACC, other variables, including ≥3 prior therapies and elevated platelets were also found to have prognostic significance; however, only 4% of the patients included in that study were sarcoma patients, and the number of bone sarcoma patients was not specified. In the present study, compared with the RMH scoring system, the MDACC system accounted for only 1 additional variable—ECOG performance status, which resulted in prognostic scores of 0-4, rather than 0-3—and thus did not add to the RMH score. In fact, it may have detracted, although not substantially. On the basis of these findings, we conclude that the RMH prognostic scoring system serves as an appropriate model to aid clinicians in the selection of bone sarcoma patients for referral to and participation in phase I clinical trials.

The present study's findings are similar to those of 2 other studies that evaluated the outcome, prognosis, and clinical benefit sarcoma patients derive from early-phase clinical trials. Jones et al. found that only elevated LDH levels and low albumin levels were significantly associated with OS duration. [17] The study included older patients (median age, 48 years) who predominantly had soft tissue sarcomas; 9% had chondrosarcomas and < 4% of patients had osteosarcomas. The study's authors acknowledged that they did not perform a subgroup analysis based on tumor histology, as the small number of patients in each group would have limited the usefulness of the findings. The RMH score was also validated in a pooled analysis of 178 sarcoma patients treated on phase I protocols. [18] This study also predominantly included patients with soft tissue sarcomas. It included only 22 patients (12.4%) with aggressive bone sarcomas, and these patients were found to have the poorest outcomes, with a median OS duration of 16.6 weeks. Similarly, we found that the median OS durations of patients with Ewing sarcoma and osteosarcoma were significantly shorter than that of patients with chondrosarcoma, which is known to be a more indolent tumor.

Determining the clinical benefit of systemic therapies in phase I trials for bone sarcoma patients poses a unique challenge. First, because these trials are designed to evaluate safety rather than efficacy and second, many sarcomas—particularly osteosarcomas and chondrosarcomas—do not demonstrate dimensional responses [19]. This characteristic limits the utility of RECIST in identifying patients who are deriving a benefit from therapy. Often, alternative means of response assessment such as PET/CT imaging which can be useful in high grade bone sarcomas are not included in these trials. As with other tumor types, subtype specific phase II trials with appropriate endpoints are needed to confirm benefit. Some experts have suggested that PFS is a more appropriate measure of clinical benefit in sarcoma patients. [20, 21] Although benchmarks for a systemic therapy's activity against soft tissue sarcomas have been suggested, similar benchmarks for activity against advanced bone sarcomas have not been established. [22] With a lack of adequate response assessment for osteosarcoma or chondrosarcoma patients included in these studies, it is difficult to conclude whether the 4-month PFS reported in our study represents clinical benefit or simply reflects the natural history of these tumor types in the advanced setting. Our findings indicate that, despite the lack of responses in osteosarcoma patients, 4-month PFS may serve as a potential baseline for assessing the anti-tumor activity of future therapies against osteosarcomas. Responses to anti-IGF-1R therapy alone or in combination with mTOR inhibition in Ewing sarcoma patients have been reported, [23] as have responses to Apo2L/TRAIL therapy in chondrosarcoma patients. [24] When responses in Ewing sarcoma patients were seen, the availability of a promising agent increased referral of patients with bone sarcomas and rare tumors to early-phase clinical trials, which led to expansions of the phase I trials and subsequent phase II trials of anti-IGF-1R therapy alone [25–27] or in combination with mTOR inhibition. [28–30]

The major limitations of this study included its inclusion of patients with late-stage cancer, the referral bias inherent to phase I trials conducted in tertiary care centers, and the fact that patients received heterogeneous therapies. However, given the paucity of new drugs for patients with these rare diseases, any effort to enroll bone sarcoma patients in novel clinical trials to identify potential new drugs against the disease is worthwhile. During the time period of this study, additional Ewing sarcoma patients were treated on phase I trials of IGF-1R therapies within the Pediatrics and Sarcoma Medical Oncology Departments within our institution; however these patients were not captured in this analysis. In addition, none of the patients in the present study were enrolled in trials of immune checkpoint inhibitors due to the time period of the study and the clinical trials available. There is significant enthusiasm for the potential efficacy of immune checkpoint inhibitors or other immunotherapies in bone sarcoma. Evaluation of these agents in the phase I and phase II setting is currently underway.

In summary, early-phase clinical trials are essential to developing therapies for patients with aggressive-biology bone sarcomas. Our findings, which validate the RMH prognostic scoring system in this group of patients, indicate that patients may derive a clinical benefit from participating in phase I trials, and that RMH prognostic scores in particular are useful in selecting bone sarcoma patients for such studies. Our findings reiterate the importance of considering bone sarcoma patients for phase I trials following the progression of their disease on standard therapy or, in the case of chondrosarcoma patients, at diagnosis if possible.
